# Genomic, Metabolic, and Immunological Characterization of GMP-Grade Mycobacterium phlei

**DOI:** 10.1128/spectrum.00070-22

**Published:** 2022-06-21

**Authors:** Tong Qiu, Guangping Luo, Jinfeng Jiang, Ping Ding, Qintong Li

**Affiliations:** a Departments of Obstetrics & Gynecology and Pediatrics, West China Second University Hospital, Key Laboratory of Birth Defects and Related Diseases of Women and Children, Ministry of Education, Development and Related Diseases of Women and Children Key Laboratory of Sichuan Province, State Key Laboratory of Biotherapy and Collaborative Innovation Center of Biotherapy, Sichuan University, Chengdu, China; b Non-Coding RNA and Drug Discovery Key Laboratory of Sichuan Province, Chengdu Medical College, Chengdu, China; University of Mississippi

**Keywords:** *Mycobacterium phlei*, *Mycobacterium bovis* BCG, genomics, metabolomics, immunity, BCG

## Abstract

Mycobacterium phlei (*M. phlei*) is an understudied microbe with medical values as an immunomodulating agent. Here, we establish an industrial strain of *M. phlei*, CUD, and characterize its genomic, metabolic, and immunological profiles. The established strain has been stably passed for more than a decade, indicated by next-generation sequencing of its 5.3 Mb genome. We show that the intramuscular inoculation of heat-inactivated CUD in immunocompetent mice is well tolerated, and can mount immunological responses. Immunophenotyping demonstrates induced innate and adaptive immune responses in peripheral blood, spleen, and inguinal lymph nodes of CUD-treated mice. Using GC-TOF-MS, we find that the metabolomic profiles of different batches are highly concordant. These results demonstrate a highly reproducible production of *M. phlei* under GMP conditions.

**IMPORTANCE** Heat-inactivated *M. phlei* demonstrates promising efficacy to treat BCG-unresponsive non-muscle-invasive bladder cancer patients in clinical trials. However, lack of GMP-grade heat-inactivated *M. phlei* hampers further clinical investigations. Here, we described a GMP-grade, heat-inactivated *M. phlei* product, and presented initial characterization of its safety and immunomodulating properties. This product will serve as a starting point for further preclinical studies as well as clinical trials such as in combination with immune checkpoint inhibitors to treat bladder cancer.

## INTRODUCTION

Mycobacterium is intertwined with human life. Among approximately 190 species in this genus, Mycobacterium bovis bacillus Calmette-Guérin (BCG) and Mycobacterium tuberculosis (*M*tb) are intensively studied (1). *M*tb has co-evolved with human host (2), and causes infectious disease tuberculosis (TB), resulting in global health burden (3). To counteract *M*tb infection, BCG is widely used as vaccine (4). Nevertheless, the efficacy of BCG vaccine is about 70% to 80% against childhood tuberculosis (5). BCG is also used as immunomodulatory agent in other contexts. For example, BCG remains the gold-standard treatment for non-muscle-invasive bladder cancer (NMIBC) for more than 40 years (6). However, despite initial response in many patients at 1 year after treatment, 75% of patients become BCG-unresponsive within 5 years resulting in high mortality (7). BCG is also used to reduce recurrent respiratory tract infections as well as acute attacks of chronic bronchitis. Other species are less studied, and medical values of mycobacterial space remain to be explored.

Mycobacterium phlei (*M. phlei*) is an understudied microbe, first isolated by Alfred Moëller in 1898 (8, 9). *M. phlei* is a nonpathogenic, nontuberculous mycobacterial species (10). Previous studies suggested that heat-inactivated *M. phlei* has antitumor activities *in vitro*, similar to BCG (11). The partial genome sequence of several strains of *M. phlei* has recently been published (12). Nevertheless, GMP-grade, industrial scale production of *M. phlei* has not been reported, limiting further exploration of its medical value.

Heat-inactivated *M. phlei* was approved about 20 years ago by the China National Medical Products Administration (NMPA, formerly known as the China Food and Drug Administration) (S20040067-70) as an agent to potentially boost human immunity. It should be noted that heat-inactivated *M. phlei* was approved as a general immunomodulating agent, but not as a drug to treat specific disease. The safety of heat-inactivated *M. phlei* in humans has been ascertained in the past 20 years. However, its mechanism-of-action as well as its efficacy on specific disease are still poorly understood. Interestingly, recent clinical studies have demonstrated the efficacy of heat-inactivated *M. phlei* to treat BCG-unresponsive NMIBC patients (11, 13). Novel treatment options for NMIBC present a major unmet medical need worldwide (14), promoting us to produce GMP-grade heat-inactivated *M. phlei* for further clinical testing. The original production and quality control processes were suboptimal due to technical limitations 20 years ago, and new guidelines from NMPA have been upgraded since then. Therefore, in the present study, we aimed to use advanced genomic and metabolic techniques to characterize an industrial strain of *M. phlei* (named CUD) as well as a GMP-grade product containing heat-inactivated CUD. It is our hope that this study will serve as a starting point to provide GMP-grade heat-inactivated *M. phlei* for ongoing clinical trials to examine its efficacy on cancer and respiratory diseases.

## RESULTS

### Genomic features of Mycobacterium phlei CUD.

**FIG 1:**
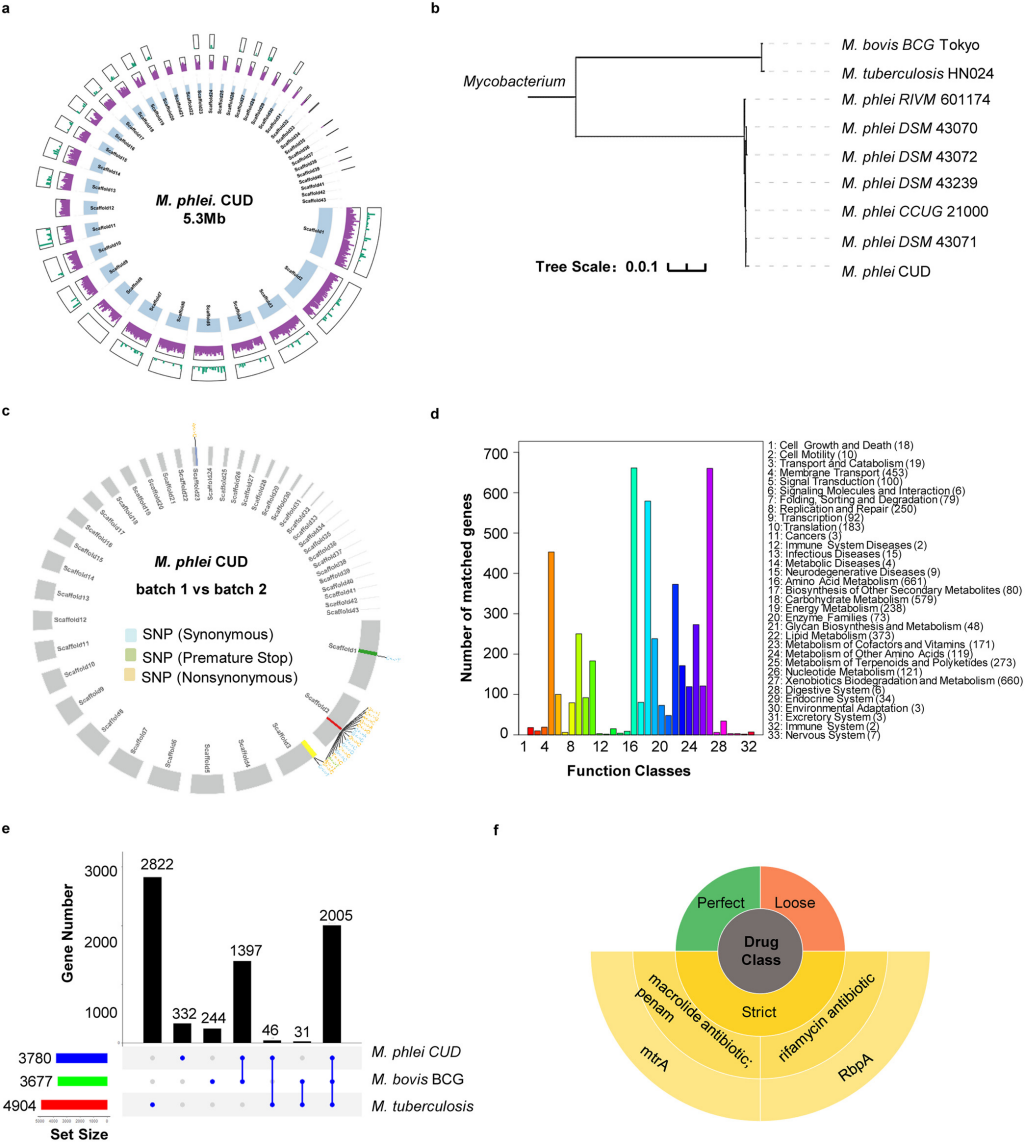
Genomic features of Mycobacterium phlei strain, CUD. (a) A circos plot of CUD genome assembly. From inner to outer circles, blue blocks denote 43 scaffolds of CUD. Purple histogram represents gene densities of each scaffold. The green histogram track shows single nucleotide polymorphisms (SNPs) compared with the whole-genome sequence of *M. phlei* CCUG21000 (NCBI reference sequence, NZ_ANBP01000001.1), containing 5,454,064 nucleotides. Of note, the assembly of CCUG21000 genome is complete, thus CUD genome assembly is ~98.4% complete (5,366,584 nucleotides). (b) Phylogenetic tree of six *M. phlei* strains as well M. tuberculosis and M. bovis BCG based on comparison of 2,000 core conserved genes calculated by core-pan analysis. Branch length represents the size of evolutionary distance. (c) A circos plot of detected variants between two batches of GMP-grade CUD. Out of 58 variants detected by second generation sequencing, 19 variants were located in putative protein-coding regions, denoted on corresponding scaffolds. Of note, these variants were localized in four genes. (d) KEGG pathway analysis of predicted protein-coding genes of CUD. (e) Analysis of homologous and divergent genes between mycobacterial species CUD, M. bovis BCG and M. tuberculosis. (f) Resistance Gene Identifier (RGI) analysis of CUD using AMR gene family, drug class and resistance mechanism based on antibiotic resistance database (CARD).

We analyzed the genome of Mycobacterium phlei CUD (CUD) by next-generation sequencing using Illumina platform (Fig. 1a). Forty-three overlapping scaffolds were generated. We found that the genome of CUD contained approximately 5.3 million base pairs (Mb) (GC content, 69.47%), close to those of *M. phlei* CCUG 21000 (5.35 Mb, NCBI Assembly: GCA_001583415.1), *M. phlei* NCTC8156 (5.31 Mb, NCBI Assembly: GCA_900453675.1) and *M. phlei* RIVM700384 (5.68 Mb, NCBI Assembly: GCA_900453675.1) (Fig. 1b).

To determine the genomic stability of CUD, different batches produced under GMP standard were subjected to next-generation sequencing (Fig. 1c). CUD has been maintained for more than 2 decades, and we compared two batches produced in 2010 (batch A) and 2020 (batch B). We found that batch A and batch B shared >99.99% identity, and only differed in 57 nucleotides out of its 5.3 Mb genome (Fig. 1c). Because > 99.99% identity exceeds the accuracy of Illumina sequencing platform (Q20), this suggests that the nuance in nucleotide composition between two batches might arise from sequencing error of Illumina platform. Thus, the genome of CUD is stable under GMP condition.

GeneMarkS was used to predict protein-coding genes of *M. phlei* CUD (15). Overall, 5,126 genes were predicted, covering 92.04% of *M. phlei* CUD genome. Among them, 3,780 genes were predicted to encode proteins. Microbial pathway enrichment analysis using the KEGG database (hsa00001) revealed that most genes were involved in metabolic pathways, including amino acid metabolism, carbohydrate metabolism as well as xenobiotics biodegradation and metabolism (Fig. 1d).

Core-pan analysis was performed to predict homologous genes between *M. phlei* CUD and M. bovis BCG and M. tuberculosis (Fig. 1e). We found that CUD, BCG and M. tuberculosis had 2,005 homologous genes. For pairwise comparison, we found that 1,397 genes of CUD had homologues in BCG but not in M. tuberculosis, and 46 genes in CUD had homologues in M. tuberculosis but not in BCG (Fig. 1e). This result suggests that CUD may impact human immune system in a similar fashion as BCG. Finally, we used RGI software in the comprehensive antibiotic resistance database (CARD) to predict potential resistance of CUD to antibiotics. Consistent with its high homology to M. bovis, BCG, and M. tuberculosis, *M. phlei* CUD was predicted to resist macrolide and rifamycin (Fig. 1f).

### Safety profile of heat-inactivated *M. phlei* CUD in immunocompetent mice.

**FIG 2:**
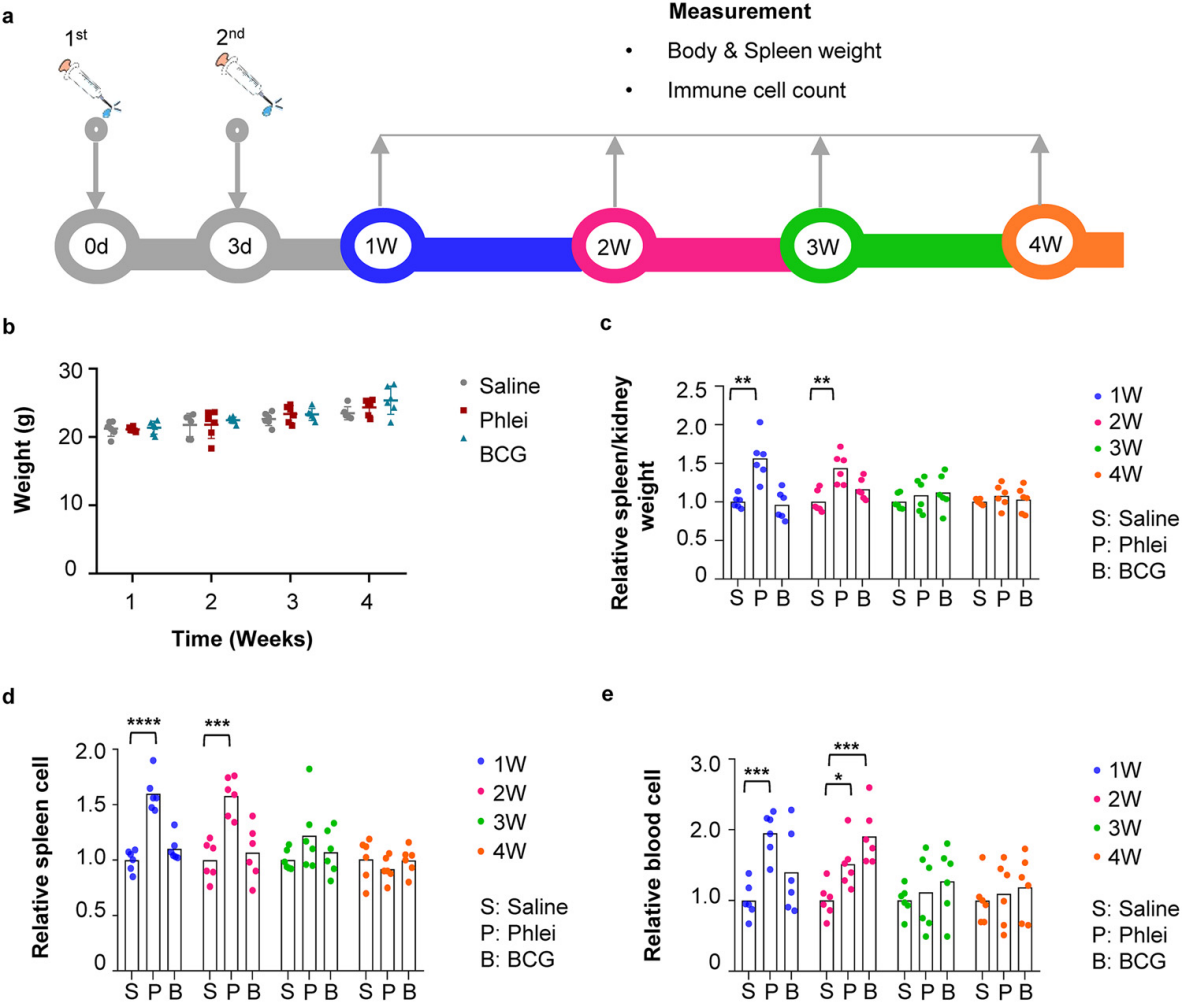
Safety profile of heat-inactivated *M. phlei* CUD in immunocompetent mice. (a) Experimental scheme. Mice were injected twice with *M. phlei* CUD and M. bovis bacillus Calmette-Guérin (BCG), individually, at day 0 and day 3, followed by measurements of body weight, spleen weight, and immune cells each week for 4 weeks. D, day; W, week. (b) Dot plot representation of body weight of mice treated with saline, *M. phlei* CUD (phlei) or M. bovis *BCG* (BCG). Data are represented as mean ± S.D. (*n* = 6). (c) Box plot representation of spleen weight of mice treated with saline, *M. phlei* CUD (phlei) or M. bovis *BCG* (BCG). Spleen weight was normalized using the weight of kidney because kidney weight was comparable between each group. Data are represented as mean ± S.D. (*n* = 6). (d) Box plot representation of total number of cells in spleen of mice treated with saline, *M. phlei* CUD (phlei), or M. bovis *BCG* (BCG). The quantity of immune cells in control mice was arbitrarily denoted as 1, and those of treatment groups were normalized to the control group. Data are represented as mean ± S.D. (*n* = 6). (e) Box plot representation of total number of immune cells in peripheral blood of mice treated with saline, *M. phlei* CUD (phlei) or M. bovis *BCG* (BCG). The quantity of immune cells in control mice was arbitrarily denoted as 1, and those of treatment groups were normalized to the control group. Data are represented as mean ± S.D. (*n* = 6). Statistical analysis (one-way ANOVA): *, *P*-value < 0.05; **, *P*-value < 0.01; ***, *P*-value < 0.001.

Six-week-old female BALB/c mice were used to examine whether CUD had immunostimulatory properties. M. bovis BCG was used as a positive control. Two doses of heat-inactivated *M. phlei* CUD or M. bovis BCG were injected at day 0 and day 3, followed by measurement of animal body weight, spleen/kidney weight, and immune cells at 1-week interval during a 4-week period (Fig. 2a). Saline was used as an injection control. Two independent experiments were carried out with three mice for each treatment group. No overt toxicity was found in mice injected with CUD or BCG, indicated by comparable body weight gain with mice injected with saline (Fig. 2b). Spleen was enlarged in mice injected with CUD at week 1 and week 2, and at week 3 recovered to a similar size as mice injected with saline. As expected, BCG also induced the enlargement of spleen, albeit at slower kinetics and with lower amplitude (Fig. 2c). Consistently, the number of lymphocytes in spleen (Fig. 2d) and immune cells in peripheral blood (Fig. 2e) were increased in CUD- or BCG-treated mice. These results demonstrate that heat-inactivated CUD is generally safe, and can stimulate immune responses in mice.

### Analyses of immunological reactions induced by *M. phlei* CUD.

**FIG 3:**
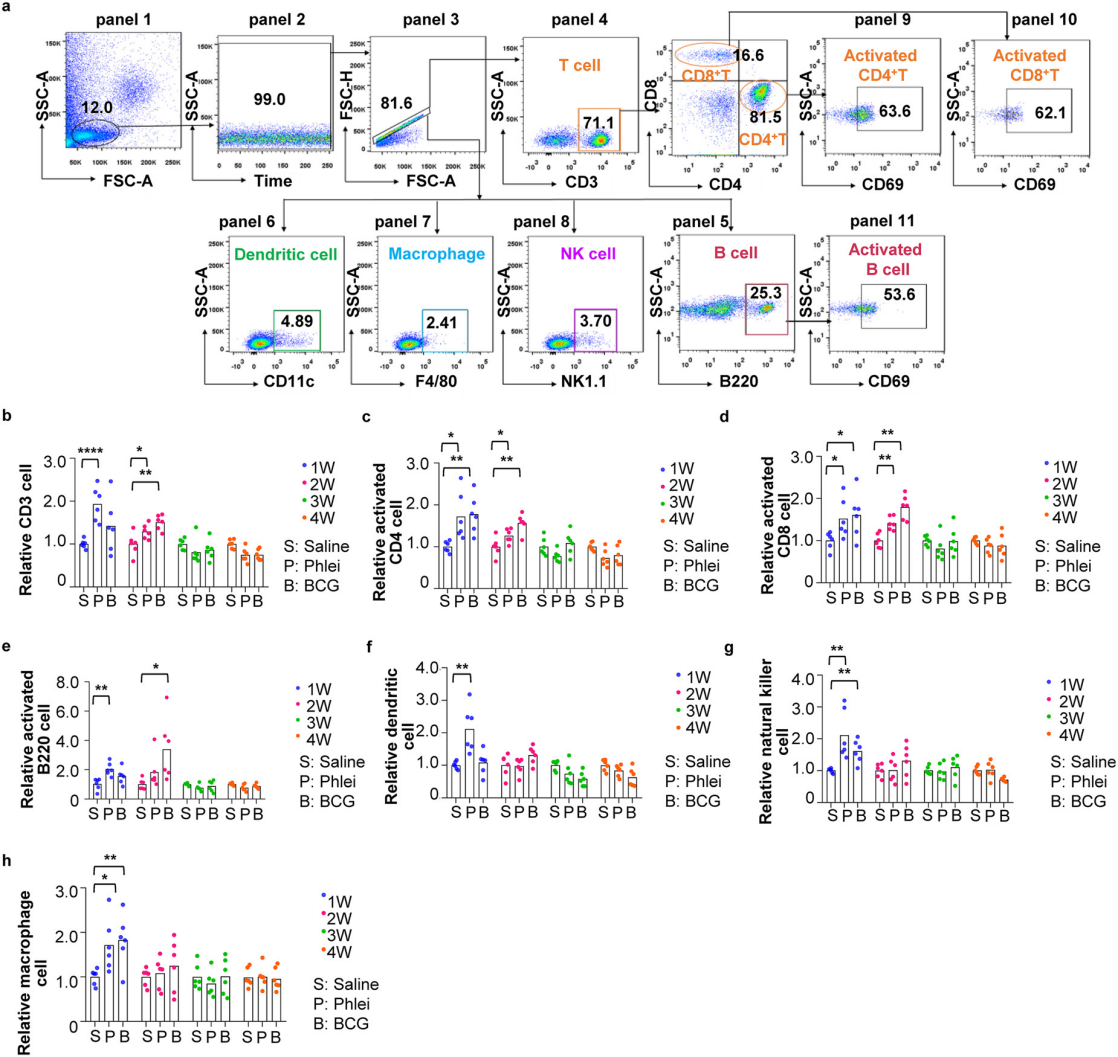
Analyses of immunological reactions in peripheral blood induced by *M. phlei* CUD. (a) Gating strategy of fluorescence-activated cell sorting (FACS) analyses to quantitate indicated cell types in peripheral blood. Lymphocytes were gated based on side scatter area (SSC-A) and forward scatter area (FSC-A) (panel 1). Time parameter was used as a quality control to exclude events bursts (panel 2), followed by gating of singlet cell (panel 3). CD3^+^ T cells (panel 4), B220^+^ B cells (panel 5), CD11c^+^ dendritic cells (panel 6), F4/80^+^ macrophage (panel 7) and NK1.1^+^ natural killer (NK) cells (panel 8) were analyzed in peripheral blood. CD3^+^ T cells were further analyzed to identify CD4^+^CD69^+^ T cells (panel 9) and CD8^+^CD69^+^T cells (panel 10). B220^+^ B cells were further analyzed to identify B220^+^CD69^+^ B cells (panel 11). (b to e) Box plot presentation of (b) total CD3^+^ T cells, (c) CD4+CD69^+^ T cells, (d) CD8^+^CD69^+^ T and (e) B220^+^CD69^+^ B cells in peripheral blood. The quantity of immune cells in control mice was arbitrarily denoted as 1, and those of treatment groups were normalized to the control group. Data are represented as mean ± S.D. (*n* = 6). (f to g) Box plot presentation of dendritic cells (f), natural killer cells (g), and macrophage (h) in peripheral blood. The quantity of immune cells in control mice was arbitrarily denoted as 1, and those of treatment groups were normalized to the control group. Data are represented as mean ± S.D. (*n* = 6). Statistical analysis (one-way ANOVA): *, *P*-value < 0.05; **, *P*-value < 0.01; ***, *P*-value < 0.001.

We further used fluorescence-activated cell sorting (FACS) to analyze subtypes of immune cells in peripheral blood, spleen, and inguinal lymph nodes. Gating strategy for peripheral blood was shown in Fig. 3a. CD3^+^ T cells were increased in CUD- and BCG-treated mice and lasted for 2 weeks after the final injection (Fig. 3b). CD69-positive CD4^+^ and CD8^+^ T cell subsets were also increased during the same time window, indicating substantial T cells were activated (Fig. 3c and d). CD69-positive B220^+^ B cells were increased for 2 weeks (Fig. 3e). Interestingly, dendritic cells, natural killer cells, and macrophages were increased in CUD-treated mice only at week 1 (Fig. 3f, g, h). These results demonstrated that CUD and BCG could stimulate quantitively similar immune reactions.

**FIG 4:**
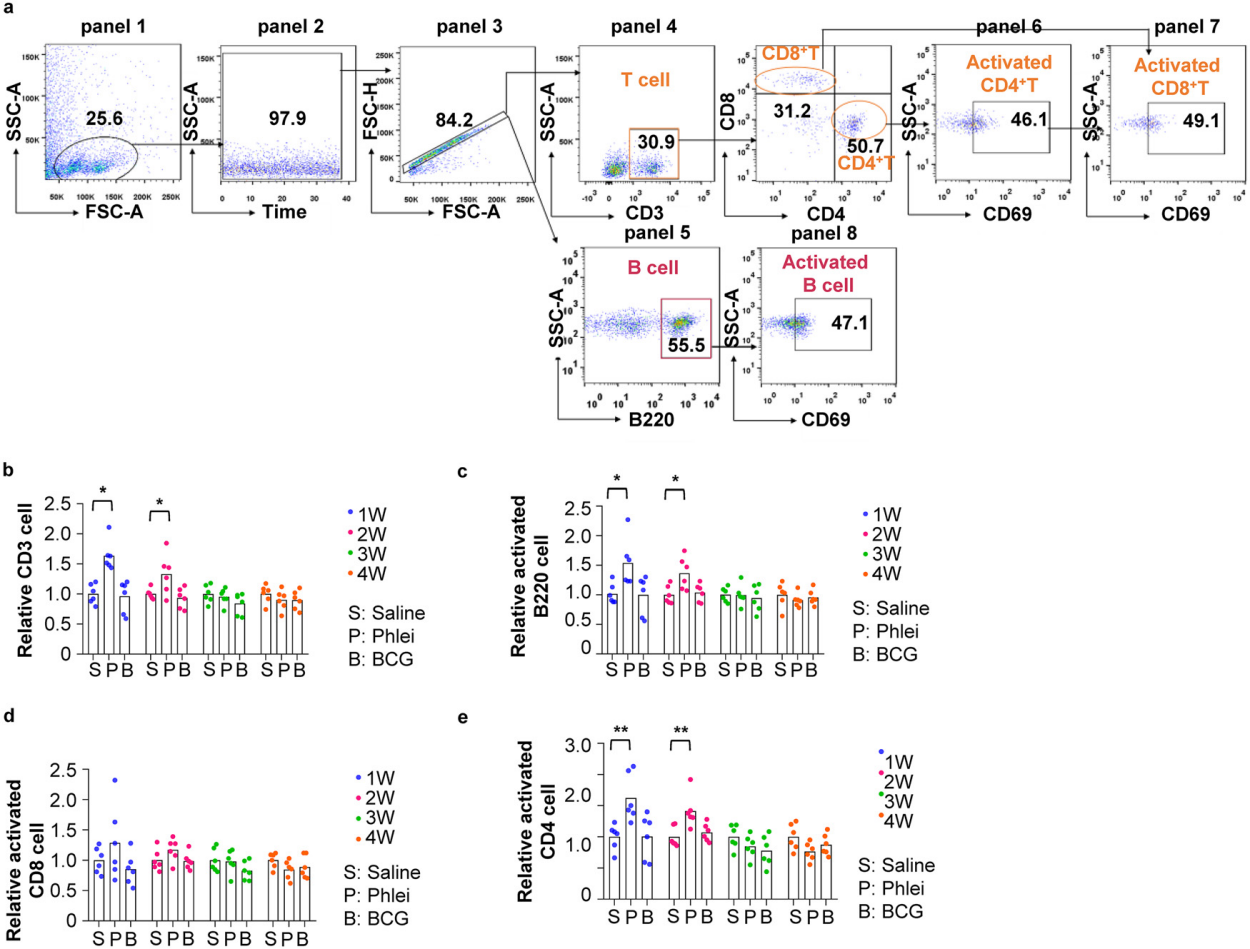
Analyses of immunological reactions in spleen induced by *M. phlei* CUD. (a) Gating strategy of fluorescence-activated cell sorting (FACS) analyses to quantitate indicated cell types in spleen. Lymphocytes were gated based on side scatter area (SSC-A) and forward scatter area (FSC-A) (panel 1). Time parameter was used as a quality control to exclude events bursts (panel 2), followed by gating of singlet cell (panel 3), CD3^+^ T cells (panel 4), B220^+^ B cells (panel 5) in spleen. CD3^+^ T cells were further analyzed to identify CD4^+^CD69^+^ T cells (panel 6) and CD8^+^CD69^+^T cells (panel 7). B220^+^ B cells were further analyzed to identify B220^+^CD69^+^ B cells (panel 8). (b to e) Box plot presentation of (b) total CD3^+^ T cells, (c) B220^+^CD69^+^ B cells, (d) CD8^+^CD69^+^ T, and (e) CD4^+^CD69^+^ T cells in spleen. The quantity of immune cells in control mice was arbitrarily denoted as 1, and those of treatment groups were normalized to the control group. Data are represented as mean ± S.D. (*n* = 6). Statistical analysis (one-way ANOVA): *, *P*-value < 0.05; **, *P*-value < 0.01; ***, *P*-value < 0.001.

FACS analyses were carried to immunophenotype cells in spleen (Fig. 4). Gating strategy was shown in Fig. 4a. The size of spleen in CUD-treated mice were increased at week 1 and 2. Accordingly, FACS analyses revealed increased T and B cells in these mice (Fig. 4b, c). However, unlike peripheral blood, only CD4^+^ but not CD8^+^ T cells were significantly activated (Fig. 4d, e).

**FIG 5:**
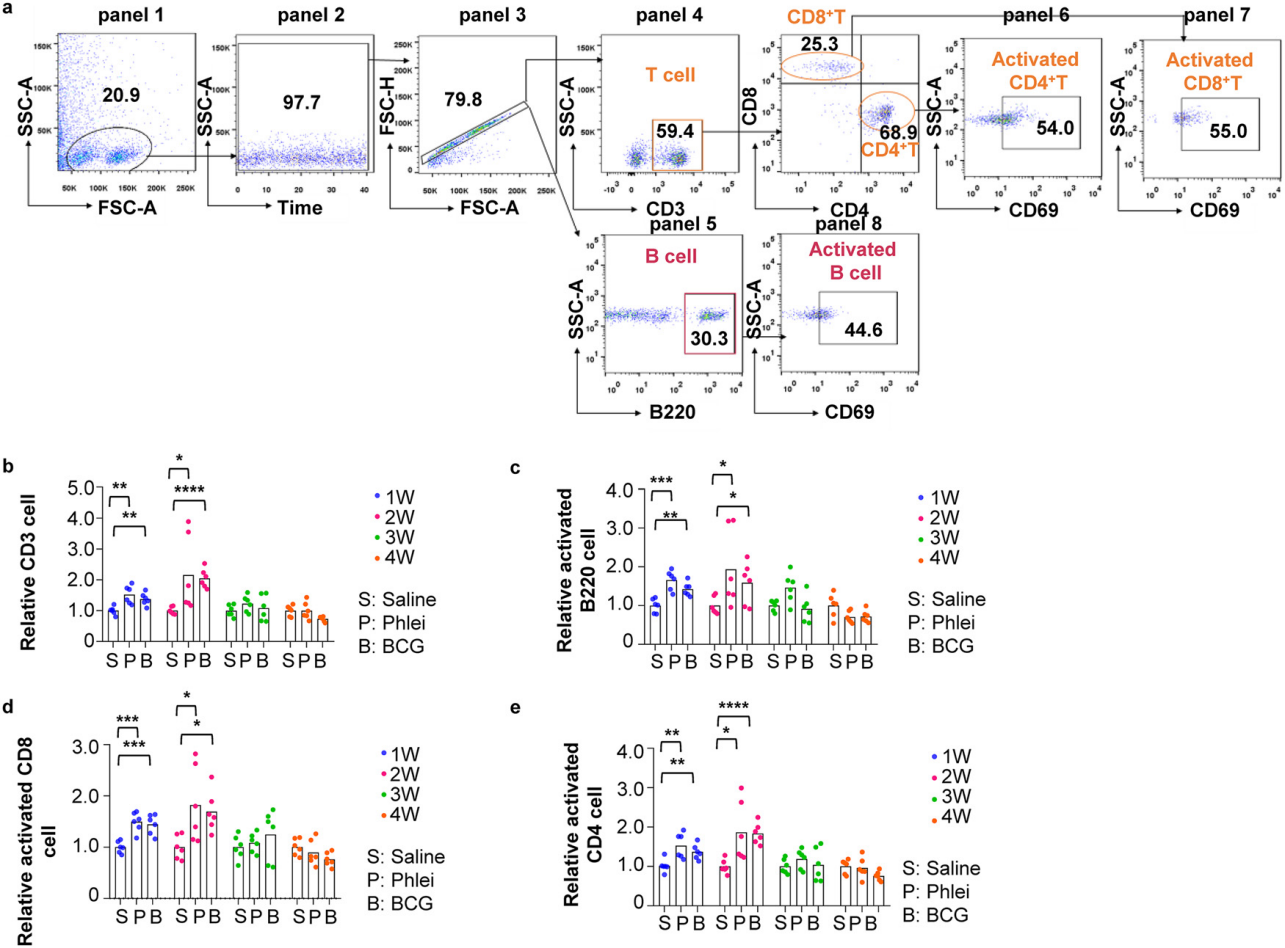
Analyses of immunological reactions in inguinal lymph node induced by *M. phlei* CUD. (a) Gating strategy of fluorescence-activated cell sorting (FACS) analyses to quantitate indicated cell types in inguinal lymph node. Lymphocytes were gated based on side scatter area (SSC-A) and forward scatter area (FSC-A) (panel 1). Time parameter was used as a quality control to exclude events bursts (panel 2), followed by gating of singlet cell (panel 3), CD3^+^ T cells (panel 4), B220^+^ B cells (panel 5) in spleen. CD3^+^ T cells were further analyzed to identify CD4^+^CD69^+^ T cells (panel 6) and CD8^+^CD69^+^T cells (panel 7). B220^+^ B cells were further analyzed to identify B220^+^CD69^+^ B cells (panel 8). (b to e) Box plot presentation of (b) total CD3^+^ T cells, (c) B220^+^CD69^+^ B cells, (d) CD8^+^CD69^+^ T, and (e) CD4^+^CD69^+^ T cells in inguinal lymph node. The quantity of immune cells in control mice was arbitrarily denoted as 1, and those of treatment groups were normalized to the control group. Data are represented as mean ± S.D. (*n* = 6). Statistical analysis (one-way ANOVA): *, *P*-value < 0.05; **, *P*-value < 0.01; ***, *P*-value < 0.001.

Finally, we used FACS analyses to quantitate immune cells in inguinal lymph nodes. Gating strategy was shown in Fig. 5a. Similar to peripheral blood (Fig. 3), numbers of total as well as activated T and B cells were increased in both CUD- and BCG-treated mice (Fig. 5b to e).

### Metabolomics analysis of six batches of GMP-grade *M. phlei* CUD.

**FIG 6:**
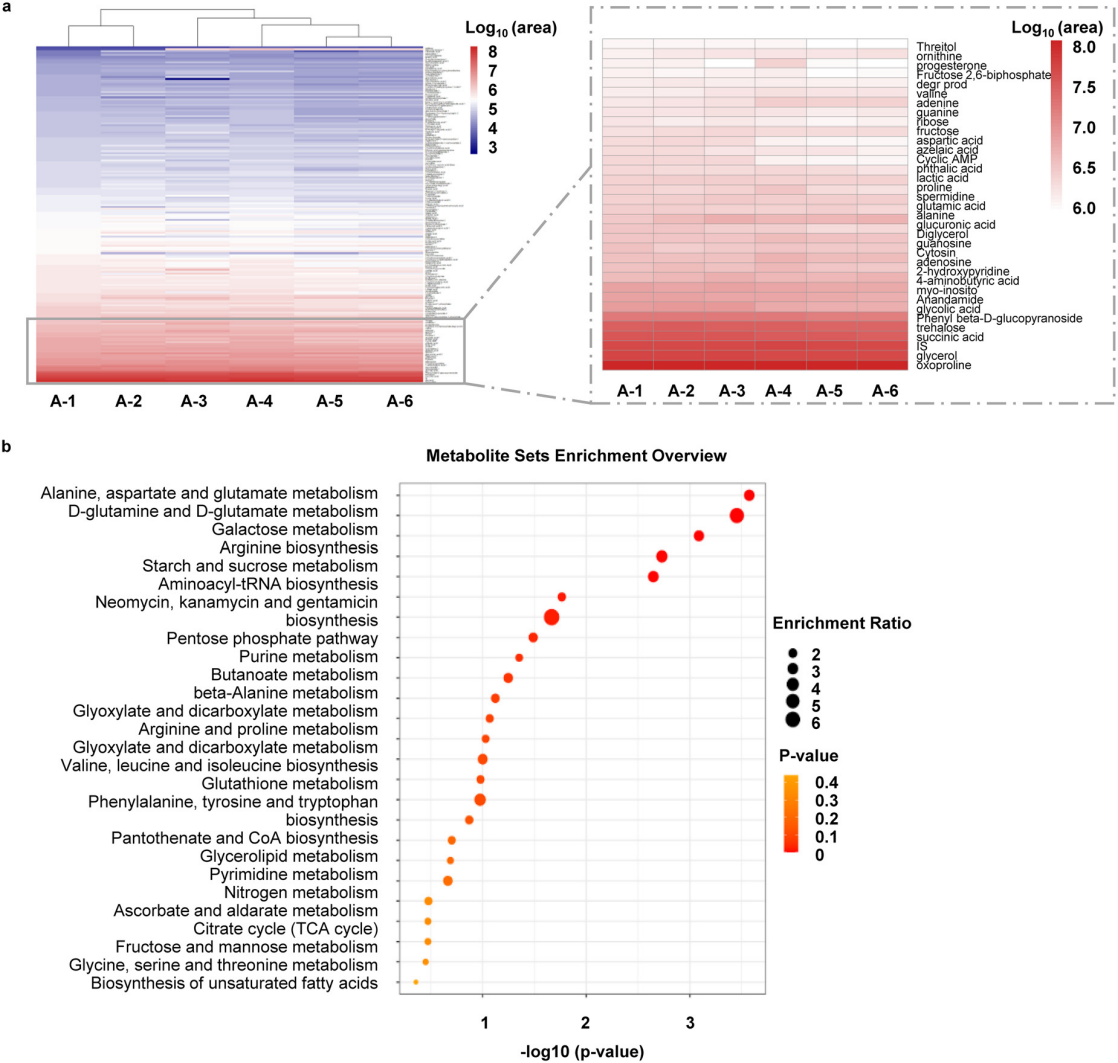
Metabolomic analysis of six batches of GMP-grade *M. phlei* CUD. (a) Heatmap presentation of 281 metabolites reproducibly identified from six batches of GMP-grade CUD and color represents relatively high and low expression, respectively. Highly expressed metabolites were listed in the dotted box. (b) Bubble chart of metabolic pathway enrichment analysis based on KEGG metabolomics database. Dot size reflected identified metabolites matched in the pathway.

To understand the immunogenic properties of *M. phlei* CUD, we further used gas chromatography time-of-flight mass spectrometry (GC-TOF-MS)-based metabolomics approach to identify metabolites generated by CUD. Six batches of CUD produced under GMP conditions were analyzed. Metabolites reproducibly identified in six batches (*t* test *P* value < 0.05, OPLS-DA VIP score >1) were calculated and aligned again KEGG (16) and HMDB databases (17). This analysis identified 281 known metabolites. Normalized peak area for each metabolite was used to estimate its abundance, and their abundance was ranked in a descending order (Fig. 6a). We also carried out metabolic pathway enrichment analysis against KEGG metabolomics database. Top 25 pathways (normalized *P* value < 0.05) are shown in Fig. 6b. These results demonstrate highly reproducible production of metabolites among different GMP-grade batches of CUD, reflecting a stable manufacturing process. Several metabolites are also of interest due to their immunomodulatory properties (see Discussion).

## DISCUSSION

*Mycobacteria* are important microorganisms due to their relevance to human health. Prominent examples include human pathogens Mycobacterium tuberculosis-causing TB and Mycobacterium leprae-causing leprosy (18). On the other hand, Mycobacterium bovis BCG is widely used in medical practice as anti-TB vaccine, immunostimulatory adjuvant, anticancer reagent, and immune modulator to reduce recurrent respiratory tract infections as well as acute attacks of chronic bronchitis (19).

Mycobacterium phlei is an understudied mycobacterial species of potential medical value. Here, we present genomic, metabolic, and immunological characterization of an industrial strain, namely, CUD, manufactured under GMP conditions. We show that different batches of CUD are genetically stable via next-generation sequencing, and produce highly reproducible metabolites via metabolic analysis. CUD product is safe and can induce immunological reactions in immunocompetent mice. A standardized, GMP-grade microbial product is a valuable tool for further clinical investigations. This is exemplified by the fact that BCG products from various vendors can exhibit differential clinical efficacy (20–22). Of note, recent clinical studies demonstrated that heat-inactivated *M. phlei* may be effective to treat BCG-unresponsive bladder cancer patients (11, 13). It will be of great interest to examine the effect of CUD in this patient population. Currently, the effect of CUD on bladder cancer is being evaluated in animal models such as the syngeneic mouse model with mouse bladder cancer MB49 cells.

In summary, we present a GMP-grade product of Mycobacterium phlei strain, CUD, which is safe and can modulate host immunity. Further studies are warranted to investigate its efficacy in the context such as cancer immunotherapy. These applications are under investigation.

## MATERIALS AND METHODS

### Genomic DNA extraction, sequencing, and assembly.

BCG was obtained from Chengdu Institute of Biological Products Co., Ltd. (S20123007, NMPA-approved agent to treat bladder cancer). *M. phlei* strain, CUD (DSM 43471), was cultured in fermentation tanks. Heat-inactivated CUD was manufactured by proprietary procedures (Chengdu Jinxing Sanum-Kehlbeck Medcine Co., Ltd.) in compliance with GMP guidelines from the China National Medical Products Administration. Genomic DNA of CUD was extracted using GeneJET Genomic DNA purification kit, analyzed by agarose gel electrophoresis, and quantified by Qubit 2.0 Fluorometer (Thermo Scientific). Sequencing libraries were prepared using NEBNext Ultra DNA Library Prep Kit for Illumina (NEB, USA), following the manufacturer’s instructions. Index codes were added to attribute sequences to each sample. Genomic DNA was fragmented by sonication to ~350 bp, and ligated with the full-length adaptor for Illumina sequencing with further PCR amplification. PCR products were purified (AMPure XP system), analyzed for size distribution by Agilent2100 Bioanalyzer, and quantified using real-time PCR. Clean data were assembled by SOAP denovo (23), SPAdes (24), ABySS (25), and Gapclose (26) to generate scaffolds with minimal gaps (Novogene, China).

### Gene prediction and annotation.

tRNA, rRNA, and sRNA were separately predicted by tRNAscan-SE, rRNAmmer and BLAST against Rfam database (27–29). Tandem Repeat Finder was used for tandem repeats analysis (30). IslandPath-DIOMB was applied in genomic islands analysis (30–34). A whole-genome BLAST search (E-value ≤ 1e-5, minimal alignment length percentage ≥ 40%) was performed against five databases: KEGG (Kyoto Encyclopedia of Genes and Genomes) (35–37), COG (Clusters of Orthologous Groups) (38, 39), NR (Non-Redundant Protein Database databases), Swiss-Prot (40), and GO (Gene Ontology) (41).

### Phylogenetic analysis.

Genomic alignment between the six *M. phlei* genomes and M. tuberculosis and M. bovis BCG in NCBI database was performed using the MUMmer (42) and LASTZ tools. Core genes and specific genes were analyzed by the CD-HIT rapid clustering of similar proteins software with a threshold of 50% pairwise identity and 0.7-length difference cutoff in amino acid (43). The phylogenetic tree was constructed by the PhyML and the setting of bootstraps was 1,000 with the orthologous genes (44). SNP, indel, and SV were found by the genomic alignment results among samples by the MUMmer and LASTZ.

### Antibiotic resistance analysis.

The Resistance Gene Identifier (RGI) was used to predict resistomes according to the genome of *M. phlei* CUD via the web portal (https://card.mcmaster.ca/analyze/rgi).

### GC-TOF-MS metabolomic analysis.

Metabolites from six batches of GMP-grade product containing heat-inactivated CUD (from > 1 × 10^7^ cells) in saline were analyzed by a commercial vendor (Biotree, China), using Agilent 7890B gas chromatography (Agilent Technologies, USA) coupled with LECO Pegasus HT/BT time-of-flight mass spectrometer (LecoCorp., USA) (GC-TOF-MS). Data, including peak numbers, sample names, and normalized peak areas, were fed to SIMCA14 software package (V14, Umetrics AB, Umea, Sweden) for principal-component analysis (PCA) and orthogonal projections to latent structures-discriminate analysis (OPLS-DA). High-resolution GC-TOF-MS spectral peak data were applied to perform metabolic pathway enrichment analysis based on KEGG metabolomics database, and a heat map was drawn using R (v3.6.3) software.

### Immunological analysis.

Animal studies were approved by the Institutional Animal Care and Use Committee at West China Second University Hospital of Sichuan University. Mice were housed under the Program of Laboratory Animal Center at West China Second University Hospital of Sichuan University with the principles and procedures of the Guide for the Care and Use of Laboratory Animals. Six-week-old female BALB/c mice were injected intramuscularly with 172 μg of heat-inactivated CUD or BCG resuspended in 500 μL saline (0.9% NaCl) per mouse, respectively. Saline was used as an injection control. The number of mice for each group was determined according to recently published guidelines (*n* = 3) (45). Two independent experiments were carried out (*n* = 6 in total for each group). Each mouse was injected on day 0 and day 3, and corresponding samples were collected on day 7, day 14, day 21, and day 28 for subsequent analyses. For fluorescence-activated cell sorting (FACS) analysis, CD3^+^ T cells, B220^+^ B cells, CD11c^+^ dendritic cells, F4/80^+^ macrophage and NK1.1^+^ natural killer (NK) cells were analyzed in peripheral blood, spleen, and inguinal lymph node. CD3^+^ T cells were further analyzed to identify CD4^+^CD69^+^ T cells and CD8^+^CD69^+^T cells. B220^+^ B cells were further analyzed to identify B220^+^CD69^+^ B cells.

### Statistical analysis.

R (v3.6.3) and GraphPad Prim 8 software were used for all statistical analyses. Statistical comparisons were made by using one-way ANOVA and Student’s *t* test. The statistical significance of difference was set as *P < *0.05. In all figures, * denotes *P* value < 0.05; ** denotes *P* value < 0.01; *** denotes *P* value < 0.001.

### Data availability.

The assembled genome sequence of CUD has been submitted to the GenBank database under the accession number PRJNA843359.
